# Promises of Protein Kinase Inhibitors in Recalcitrant Small-Cell Lung Cancer: Recent Scenario and Future Possibilities

**DOI:** 10.3390/cancers16050963

**Published:** 2024-02-27

**Authors:** Aniket Tiwari, Beauty Kumari, Srividhya Nandagopal, Amit Mishra, Kamla Kant Shukla, Ashok Kumar, Naveen Dutt, Dinesh Kumar Ahirwar

**Affiliations:** 1 Department of Bioscience & Bioengineering, Indian Institute of Technology Jodhpur, Jodhpur 342030, Rajasthan, India; 2 Department of Biochemistry, All India Institute of Medical Sciences Jodhpur, Jodhpur 342005, Rajasthan, India; 3 Department of Biochemistry, All India Institute of Medical Sciences (AIIMS) Bhopal, Saket Nagar, Bhopal 462020, Madhya Pradesh, India; 4 Department of Pulmonary Medicine, All India Institute of Medical Sciences Jodhpur, Jodhpur 342005, Rajasthan, India

**Keywords:** small-cell lung cancer, kinase, tumor microenvironment, immune checkpoint inhibitor, immunotherapy, recalcitrant, drug repurposing

## Abstract

**Simple Summary:**

The heterogeneous expression of signaling molecules within the tumor, including kinases, is the major contributor to the acquisition of drug resistance and poor survival observed in small-cell lung cancer (SCLC). The addition of immunotherapy to chemotherapy has only marginally prolonged survival in patients with extensive-stage SCLC (ES-SCLC). Recent clinical trials have combined immunotherapy with the pharmacological inhibitors of kinases often dysregulated in SCLC. However, the regime has not been effective in the long term. Here, we review studies and clinical trials exploring dysregulated kinases in SCLC progression and resistance to chemotherapies and immunotherapies. We also discuss the possibility of repurposing kinase inhibitors against SCLC that have already demonstrated promising results for other types of cancers.

**Abstract:**

SCLC is refractory to conventional therapies; targeted therapies and immunological checkpoint inhibitor (ICI) molecules have prolonged survival only marginally. In addition, ICIs help only a subgroup of SCLC patients. Different types of kinases play pivotal roles in therapeutics-driven cellular functions. Therefore, there is a significant need to understand the roles of kinases in regulating therapeutic responses, acknowledge the existing knowledge gaps, and discuss future directions for improved therapeutics for recalcitrant SCLC. Here, we extensively review the effect of dysregulated kinases in SCLC. We further discuss the pharmacological inhibitors of kinases used in targeted therapies for recalcitrant SCLC. We also describe the role of kinases in the ICI-mediated activation of antitumor immune responses. Finally, we summarize the clinical trials evaluating the potential of kinase inhibitors and ICIs. This review overviews dysregulated kinases in SCLC and summarizes their potential as targeted therapeutic agents. We also discuss their clinical efficacy in enhancing anticancer responses mediated by ICIs.

## 1. Introduction

Lung cancer (LC) is the leading cause of cancer-related deaths worldwide in both men and women [1]. The Global Cancer Observatory (GLOBOCAN) data for the year 2020 recorded 2,206,771 new incidences of lung cancer [2]. The incidence rate is similar between males and females, and it is the second-most prevalent cancer after breast cancer [3]. Although LC is the second most prevalent cancer, its death rate is the highest among all cancers [4]. About 1,796,144 lung cancer-related deaths were recorded in the year 2020 [2]. A variety of factors contribute to the development and progression of lung cancer, including genetic alterations, epigenetic regulation, environmental factors, pre-existing medical conditions, and lifestyle factors. Among various lifestyle factors, tobacco smoking remains the most significant risk factor involved [5]. Lung cancer is often diagnosed at the advanced stage. Most patients are already positive for metastasis at first diagnosis. The reasons behind the late diagnosis of lung cancer include the lack of any symptoms during the early stage of lung cancer and the inability of routine imaging techniques to detect lung cancer [6]. Lung cancer is divided into two broad subtypes: non-small cell lung cancer (NSCLC) and small-cell lung cancer (SCLC). NSCLC accounts for 85% of lung cancer cases, whereas SCLC occurs in the rest of the 15% of lung cancer patients. The classification of SCLC subtype has been determined through clinicopathological analysis, identifying two distinct stages: limited-stage SCLC (LS-SCLC) and extensive-stage SCLC (ES-SCLC). The LS-SCLC condition is limited to one lung and the nearby lymph nodes. Overall, less than one-third of patients are diagnosed with LS-SCLC [7]. The rest of the SCLC cases are ES-SCLC, which is characterized by the spread of cancer cells in both the lungs and distant organs. However, modern SCLC classification follows TNM (tumor, node and metastasis) staging guidelines, which classifies patients based on the morphology of tumor cells, invasion stage and location of metastasis [8].

Primary lung cancers commonly spread to other organs, a process known as metastasis, to the brain, bones, liver, and adrenal glands [9]. The metastasis rate differs for different subtypes of lung cancer, with the highest metastasis rate reported in SCLC patients [9]. Most SCLC cases are diagnosed with metastatic SCLC, which makes systemic chemotherapy the recommended treatment for patients. Chemotherapy-treated SCLC patients show relapse after a short period, resulting in 5-years overall survival (OS) in only 2 percent of patients. Immunotherapy with the chemotherapy regime has improved survival, but only marginally [10,11,12,13]. This might be due to the lack of cytotoxic T cells in the immune microenvironment of SCLC cells, as immunotherapy works by activating T cells against the cancer cells. Therefore, a dire need exists to develop novel therapeutic agents targeting SCLC cells more effectively, improving patient survival.

Kinases are a constituent of a heterogeneous group of enzymes called phosphotransferases. These enzymes are responsible for the phosphorylation of target proteins involved in different biological processes, including proliferation, cell cycle progression, apoptosis, cellular motility, growth, and differentiation. Any deviation from the typical functioning of these kinases can lead to different types of diseases, including cancer [14]. Specifically in SCLC, kinases are aberrantly expressed in signaling pathways, such as PI3K (phosphoinositide 3-kinase)–AKT–mTOR (mammalian target of rapamycin), which aids in SCLC cell survival and chemotherapy resistance [15]. This suggests their pivotal role in priming SCLC cells for drug resistance [16,17,18]. They participate in canonical signaling pathways in normal human body cells and regulate cellular functions. However, in cancer cells, these kinases are dysregulated via different mechanisms, as discussed in the section ahead. Some kinases altered in SCLC and other cancers are Chk1 (checkpoint kinase 1), WEE1 (nuclear kinase), PARP (poly (ADP-ribose) polymerase), and AURK (Aurora kinase). Recent advances in genomic, proteomic, and bioinformatic technologies have allowed for the development of anticancer pharmacological inhibitors specific to molecular targets, especially against hyperactive kinases. Although various kinase inhibitors have shown promising results in NSCLC, only a few are available for SCLC patients. Currently, kinases involved in cell cycle regulation and DNA repair pathways, i.e., WEE1 inhibitor (Adavosertib, also known as AZD1775, manufactured by AstraZeneca, Cambridge, UK), CHK1 inhibitor (Prexasertib, also known as LY2606368, manufactured by Eli Lilly, Indianapolis, IND, USA), and CDK4/6 inhibitor (Trilaciclib, also known as G1T28, manufactured by G1 Therapeutics, NC, USA), are under clinical investigation. These inhibitors have also not significantly improved the survival of SCLC patients significantly.

Protein kinases regulate not only chemotherapy responses but immunotherapy responses as well. The United States Food and Drug Administration (U.S. FDA) has recently approved an ICI (Atezolizumab, an anti-PD-L1 antibody, manufactured by Genentech, San Francisco, CA, United States) in combination with first-line chemotherapy for SCLC treatment. However, the benefits remain only marginal [10,11,12,13]. Subsequent studies have defined the critical role of kinases in regulating ICI-induced immune responses [19,20]. Based on this observation, combining an ICI with a WEE1 inhibitor (i.e., AZD1775) showed an improved ability of the ICI to target SCLC [19]. 

Since kinase inhibitors have been shown to sensitize recalcitrant SCLC for chemotherapy and immunotherapy, they might be developed as a combination therapy to achieve better clinical responses and longer patient survival. In this review, we summarize the kinases that are abruptly expressed or activated during the progression of SCLC and the acquisition of resistance against different therapies, including chemotherapy and immunotherapy. We also review the recent efforts to repurpose kinase inhibitors against SCLC.

## 2. Origin and Progression of SCLC 

Lungs consist of a diverse array of discrete cellular components. Understanding the cellular components implicated in the onset of SCLC and its pathological traits is significant. The initial research conducted revealed antigens specific to macrophages on SCLC cell lines and suggested that SCLC may originate from macrophages in the bone marrow [21]. Subsequently, one study conducted established the presence of lymphoreticular antigens in numerous SCLC cell lines [22]. The antigens encompass Leu-7, which is found on natural killer (NK) cells and macrophages, Leu-M1, which is present on macrophages and neutrophils, and Leu M2, which is expressed on macrophages. However, sufficient evidence was not available about the cell type involved in the origin of SCLC. A clear understanding of the cell type from which SCLC originates, came after the identification of universal biallelic deletion of *Rb* and *TP53* genes in SCLC patients and the development of a genetically engineered mouse model (GEMM) harboring the loss of *Rb* and *TP53* genes in a cell-specific manner. Using a conditional knockout (KO) of *Rb* and *TP53* in different types of cells in the lung, it was identified that the CGRP (calcitonin gene-related peptide)-expressing NE cells are the source of SCLC initiation [23]. Multiple studies utilizing whole genome-sequencing have shown evidence of the inactivation of the *Rb* and *TP53* genes in tumors of SCLC. Antone Burns’ team devised a mouse model (*RP* mouse model) within which the conditional deletion of *Rb* and *TP53* could be achieved in a cell-specific way, as indicated by the aforementioned observation [24]. This study revealed that the removal of *Rb* and *TP53* genes in pulmonary neuroendocrine cells (PNECs) and alveolar type II (ATII) cells expressing surfactant protein C (SPC) serves as the initial trigger for the development of SCLC tumors [23]. Park et al. conducted a subsequent investigation to validate the NE source of SCLC in the *Rb/TP53*-conditional-deletion mouse model [25]. The PNEC is an infrequent cellular subtype found inside the lungs, displaying characteristics associated with neurosensory functions, secretory activity, and stem cell-like traits [26,27,28,29]. Previous investigations utilizing lineage tracing techniques have shown evidence that a subset of PNEC is responsible for initiating SCLC [30]. The process of inducing the differentiation of human embryonic stem cells (HESCs) into PNECs, while concurrently suppressing the *Rb* and *TP53* genes, resulted in the initiation of early-stage SCLC upon transplantation into immunocompromised mice.

Furthermore, next-generation sequencing (NGS) investigations have revealed the presence of supplementary driver genes in primary tumors of SCLC, with the universal biallelic inactivation of *Rb* and *TP53* genes [31,32,33]. The application of comprehensive genomic methodology has successfully identified the presence of frequently mutated genes, including *CREBBP* (15–17%), *Rbl2*, *MYC*, *EP300*, *FRFR1*, and *PLCG2*, in tumors of patients diagnosed with SCLC.

To characterize these identified driver genes, many different labs have mutated them in SCLC transgenic mouse models. Jia et al. developed a triple KO mouse model of SCLC by deleting *Rb/TP53* and *CREBBP* (also known as *CBP* or *KAT2A*) in the lungs [34]. This study established the tumor suppressive role of *CREBBP* in SCLC. In terms of molecular mechanisms, the analysis of gene expression revealed that the inactivation of *Crebbp* led to a decrease in the expression of tight junction-related genes and cell adhesion, such as *Cdh1* (cadherin 1). Conversely, the suppression of *Cdh1* facilitated SCLC transformation [34]. The silencing of *Crebbp* resulted in decreased histone acetylation in *CDH1* and other adhesion genes. The administration of Pracinostat, an inhibitor of histone deacetylase (HDAC), resulted in elevated levels of histone acetylation and the subsequent re-establishment of *CDH1* gene expression [34]. 

Besides *CREBBP*, comprehensive genomics have also identified another frequently mutated gene, *EP300* (5–13%) (also called *P300* or *KAT2B*), an acetyltransferase, like *CREBBP* [31]. Both CREBBP and EP300 have alike domain components and also share high sequence homology [35]. The involvement of EP300 in the growth of SCLC was determined through the utilization of an SCLC transgenic mice that lacked the *EP300* gene [36]. Mutations in the HAT (histone acetyltransferase) domain of EP300 are frequently observed [24]. The inhibition of this domain hinders the progression of SCLC, while its deactivation facilitates the growth of SCLC. Moreover, after the dissection of EP300, it was shown that it encompasses three additional domains: KIX (kinase-inducible domain-interacting), BAD (bromodomain), and TAZ (transcriptional adaptor zinc-binding). These domains have been found to interact with transcription factors, notably *MYB*, and are considered to be the key factor responsible for the pro-tumorigenic activity observed in SCLC [36].

A recent study employed an SCLC GEMM known as *Rb^fl/fl^*; *TP53^fl/fl^*; *Rbl2^fl/fl^* (referred to as *RPR2*) mouse model to characterize the role of *Rbl2* (or *p130*) [37]. The candidates that were found had an enrichment in pathways associated with oncogenic signaling, particularly in variables that have been involved in the PI3K/AKT/mTOR signaling pathways which were identified using Tuba-sequencing or Tuba-seq (tumor barcoding with ultradeep barcode sequencing). The advent of Tuba-seq has facilitated the quantitative and scalable examination of pools of potential driver genes in mouse models of lung adenocarcinoma (LADC) for functional analysis [38,39]. The present investigation has successfully identified several novel oncogenic drivers and tumor suppressors specific to SCLC, including *Tsc1* (tuberous sclerosis complex 1). 

In another study to identify SCLC driver genes, CRISPR-Cas9 technology was employed to KO the *Tsc1* gene in the *RPR2* mouse model of SCLC. This study reported that the inactivation of *Tsc1* results in the augmentation of both the frequency and size of tumors, suggesting that *Tsc1* functions as a potent tumor-suppressor gene linked to the PI3K/AKT/mTOR pathway in SCLC [33]. The PI3K/AKT/mTOR pathway contributes to the regulation of various cellular processes, including cell proliferation [40,41]. The researchers found the activation of mTORC1 and the subsequent development of giant cell carcinoma of the lung following the inactivation of *Tsc1* in mouse models of SCLC.

To characterize the role of *MYC* in SCLC, the *Rb/TP53/Myc*-overexpression (*RPM*) mouse model was developed, which represents *MYC*-overexpressing NE-low SCLC with *NEUROD1* expression [42]. *MYC* amplification contributes to the rapid progression of SCLC. The survival rates of patients with *MYC* overexpression is much lower in comparison to those with less *MYC* expression [42,43]. The GEMM *RPM* driven by *MYC* exhibits the essential characteristics of human SCLC. It is noteworthy that this model exhibits similarities to a specific subtype of human SCLC in terms of its “variant” form, elevated *NEUROD1* levels, and reduced expression of NE genes, like *ASCL1*.

*Fgfr1* has been identified as another driver gene in SCLC. A study by Kim et al. has demonstrated the tumor promoting role of FGFR1 (fibroblast growth factor receptor 1) by showing that the loss of driver genes *Rb*, *TP53* and *Rbl2* promotes SCLC growth by overexpressing *Fgfr1* [44]. Use of the pan-FGFR inhibitor PD173074 has been observed to block the proliferation of SCLC cells and prevents FGF-2 (ligand for FGFR1)-induced chemoresistance [45]. A parallel study indicated that the excessive expression of *Fgfr1^K656E^*, which represents a constitutively active form of FGFR1, in NE cells that express CGRP^POS^ (calcitonin gene-related peptide-positive; cells of the origin of SCLC) and lack functional *Rb/TP53* signaling, leads to the suppression of tumor initiation. However, *Fgfr1^K656E^* overexpression promotes SCLC cancers from tracheobronchial-basal cells [46]. 

**Figure 1:**
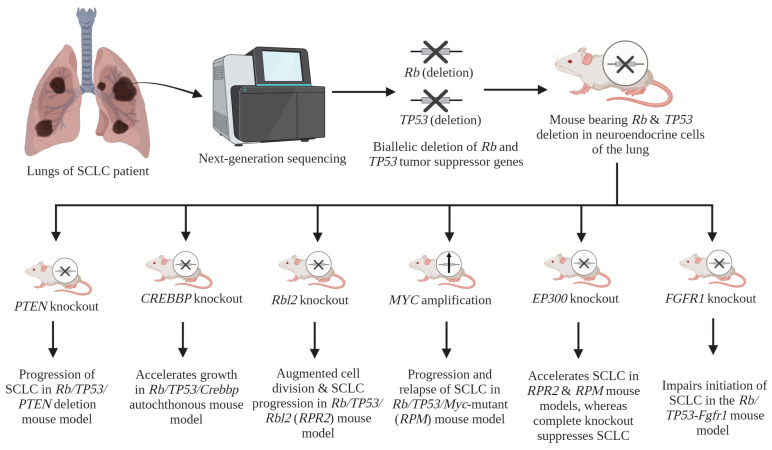
SCLC driver genes. NGS analysis of SCLC patient samples identified biallelic inactivation of the *Rb* and *TP53* genes. This knowledge was used to generate a GEMM lacking *Rb* and *TP53* specifically in the lungs. Furthermore, the model was used to define the role of additional driver genes in SCLC progression and metastasis. [Created with BioRender.com (accessed on 18 February 2024)].

Collectively, these investigations have substantiated the notion that the driver gene mutations identified in individuals with SCLC have a role in the advancement of the disease through several pathways. The driver genes of SCLC have been summarized in Figure 1 and Table 1 below.

## 3. Clinical Features of SCLC

Histologically, SCLC is a neuroendocrine (NE) tumor defined by the presence of densely packed neurosecretory granules, which are vesicles that store NE hormones. Most SCLC instances originate within the principle and secondary bronchi, which are the larger airways. Currently, a significant proportion, around 60–70%, of individuals exhibit a substantial extent of disease that is not amenable to treatment using a solitary radiation therapy approach. The median survival (MS) and OS rates of individuals diagnosed with SCLC have not changed in the last four decades. The 5-year survival rates have exhibited an upward trend, rising from 4.9% over the period spanning from 1983 to 1993 to 6.4% from 2002 to 2012. 

SCLC is commonly treated using a dual chemotherapy approach. Chemotherapy is considered the principal therapeutic approach for individuals diagnosed with SCLC because of the hard-to-reach anatomical location and the occurrence of early and widespread metastases. Other therapeutic options that may be utilized include radiation therapy or a combination of ICI-based drugs. Even though chemotherapy is successful in targeting SCLC cells, it is important to acknowledge that these cells utilize other mechanisms for cell survival and proliferation to dodge the cytotoxic effects of chemotherapy. Consequently, the effectiveness of chemotherapies and targeted medicines, whether administered alone or in conjunction, has not exhibited substantial potential for the treatment of SCLC. The investigation of ICIs has also been conducted in the quest for enhanced treatment alternatives. However, the rate of OS in patients shows only marginal changes. Kinases have a pre-eminent role not only in the regulation of the chemotherapeutic response but also in the modulation of treatment-induced immune responses. Therefore, medical professionals are presently examining the possible advantages of integrating different kinase inhibitors with ICIs to augment patients’ OS rates. Therefore, the application of inhibitors for the purpose of targeting these kinases has exhibited the capacity to augment the efficacy of treatment-resistant SCLC in response to both chemotherapy and immunotherapy.

## 4. Immunotherapy in SCLC

In conjunction with the administration of chemotherapy and targeted therapy, there have been recent endeavors to stimulate the response of the immune system to SCLC. The high tumor mutational burden (TMB) observed in SCLC has been suggested as a mechanism for generating neoantigens, which in turn may lead to a more robust immune response against the tumor. Nevertheless, SCLC tumors exhibit immune-inert characteristics. The suppression of anti-tumor immune responses is mostly attributed to two key mechanisms: reduced antigen presentation and the upregulation of ICs [49]. Numerous ICIs have been designed to overcome the immune suppression observed in solid tumors, including SCLC. ICIs disrupt interactions between the ICs present on tumor cells and their corresponding targets localized on immune cells. 

Considering the high TMB in SCLC, the rationale of combining chemotherapy with ICIs has been tested in many clinical trials [10,11,12,13,50,51,52,53,54]. The inhibition of PD-L1 (programmed cell death ligand 1 (or CD274)/PD-1 (programmed cell death 1) (or CD279) signaling has demonstrated potential in the therapeutic approach to ES-SCLC by augmenting tumor-specific T-cell immunity [10,11,12,13]. In the context of SCLC, it has been observed that PD-L1 is expressed in different cellular compartments of SCLC tissues, including NE marker-positive tumor cells [55,56]. The anti-PD-L1 antibody Atezolizumab was first evaluated in combination with carboplatin–etoposide chemotherapy in a phase III IMpower 133 clinical trial (NCT02763579) [10,11,12,13]. The findings of this study suggest that the inclusion of Atezolizumab alongside chemotherapy as a primary treatment for ES-SCLC led to a modest increase of 2 months in OS and 0.9 months in progression-free survival (PFS) compared to chemotherapy alone. This trial is a significant milestone, as it provides the initial evidence of the therapeutic advantage associated with PD-L1 inhibitors in individuals diagnosed with ES-SCLC, albeit with only a modest effect size [57]. The randomized distribution of patients without the knowledge of drug target levels might be a reason for the limited clinical response observed in IMpower133 [10,11,12,13]. Another randomized, controlled and open-label trial conducted in a phase III trial (CASPIAN, NCT03043872) evaluated Durvalumab (anti-PD-L1 antibody, manufactured by Medimmune/AstraZeneca, Gaithersburg, MD, USA) in combination with standard chemotherapy [50,51]. The trial concluded that the first line Durvalumab plus chemotherapy significantly prolonged the OS in ES-SCLC patients by 2.7 months versus the control (or vehicle). Another randomized, controlled and double-blind trial conducted in phase III (KEYNOTE-604, NCT03066778) evaluated Pembrolizumab (anti-PD-1 antibody, manufactured by Merck & Co., Rahway, NJ, USA) in concert with standard chemotherapy [52]. Pembrolizumab plus chemotherapy significantly extended the PFS and OS by 0.2 and 1.1 months, respectively, compared to the placebo. Recently, the CAPSTONE-1 trial was conducted to evaluate the efficacy of Adebrelimab (anti-PD-L1 antibody, manufactured by Jiangsu Hengrui Medicine Co Ltd., Lianyungang, China) in concert with standard chemotherapy. This trial followed a randomized, placebo-controlled and double-blind design (NCT03711305) [53]. The addition of Adebrelimab to standard chemotherapy demonstrated a statistically significant improvement in the OS by 2.5 months among patients diagnosed with ES-SCLC. Another randomized, worldwide, phase III clinical trial took place in 2022 (ASTRUM-005 NCT04063163) evaluating Serplulimab (anti-PD-1 antibody, manufactured by Shanghai Henlius Biotech, Shanghai, China) in combination with standard chemotherapy [54]. The combination of Serplulimab and chemotherapy demonstrated a notable extension of the OS among patients diagnosed with extensive SCLC, with an increase of 4.5 months. 

**Figure 2:**
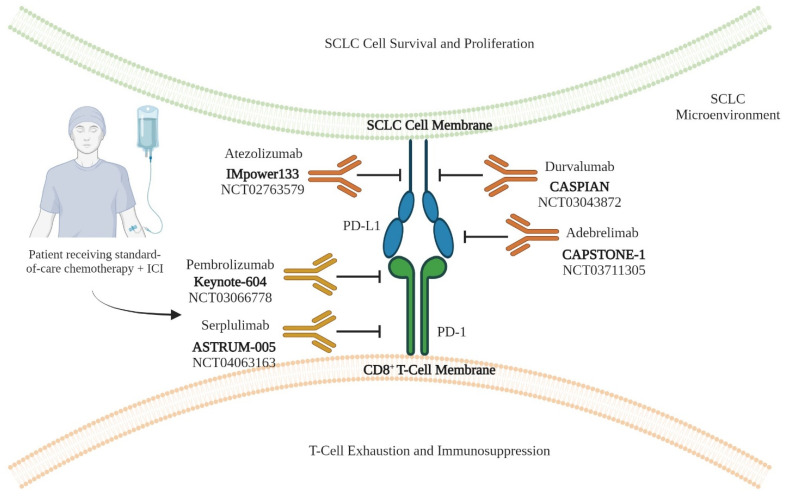
Immune checkpoint (IC) blockade and related clinical trials in SCLC. PD-L1 expressed on cancer cells interacts with PD-1 on T cells to activate exhaustion and suppression pathways in T cells. Immune checkpoint inhibitors (ICIs) that impede this interaction have been developed and evaluated in different clinical trials for treatment-naïve ES-SCLC, combined with standard-of-care chemotherapy. The clinical trials include IMpower133 evaluating Atezolizumab (anti-PD-L1 antibody), CASPIAN evaluating Durvalumab (anti-PD-L1 antibody), Keynote-604 evaluating Pembrolizumab (anti-PD-1 antibody), CAPSTONE-1 evaluating Adebrelimab (anti-PD-L1 antibody) and ASTRUM-005 evaluating Serplulimab (anti-PD-1 antibody). [Created with BioRender.com (accessed on 18 February 2024)].

Among the anti-PD-L1 antibodies evaluated so far, the OS in the CAPSTONE-1 trial was found to be longer in both groups compared to the IMpower133 trial and the CASPIAN trial. This difference may be attributed to a higher percentage of patients receiving subsequent systemic treatments in the CAPSTONE-1 trial (59% and 70%) compared to the other two trials (50.2% and 57.4% in the IMpower133 trial; 42% and 44% in the CASPIAN trial) [10,51,53]. However, the use of anti-PD-1 antibody has provided the longest OS in treatment-naïve ES-SCLC patients. A possible explanation for this observation is that the expression of PD-1 on tumor-associated macrophages (TAMs) makes them pro-tumorigenic [58,59]. The summary of all the ICI clinical trials is presented in Figure 2 and Table 2.

## 5. Role of Protein Kinases in Immunotherapy for SCLC

### 5.1. CDK4/6

Cyclin-dependent kinase 4/6 (CDK4/6) demonstrates a significant role in regulating the cell cycle in both normal and cancerous cells. Hence, the inhibition of CDK4/6 can result in the cessation of cell cycle progression. CDK4/6 inhibitors have received regulatory approval for the treatment of breast cancer and are currently undergoing clinical trials for the management of other malignant conditions, including SCLC.

A selective inhibitor of CDK4/6, Trilaciclib (C_24_H_30_N_8_O, molecular weight: 446.55) was developed using a structure-based drug-designing approach to selectively target Cyclin D-CDK4/6. It was found to selectively and reversibly inhibit the cell cycle in a CDK4/6-dependent manner [60]. Based on this activity, Trilaciclib has been shown to protect hematopoietic cells from chemotherapy treatment by arresting them in the G1 phase of the cell cycle. *Rb*-deficient cells were resistant to Trilaciclib. The G1-to-S phase of cell cycle progression in *Rb*-null SCLC cells is CDK4/6-independent. Therefore, Trilaciclib protects bone marrow cells from chemotherapy, without interfering with the ability of chemotherapy to target SCLC cells [60]. An *Rb*-null SCLC xenograft mouse model study showed that Topotecan in combination with Trilaciclib shows better antitumor effects than Topotecan alone [60]. Moreover, in SCLC patients, during chemotherapy treatment, Trilaciclib was reported to elevate the peripheral lymphocyte count and enhanced T cell activation [61]. However, Trilaciclib poses adverse hematologic effects, such as anemia, in some patients (NCT03041311) [62].

This strategy involves the utilization of alternative mechanisms that do not directly interact with immune cells, yet nonetheless have the capability to influence the immune response. The concept of combining chemotherapy and ICIs is similarly founded upon this notion. In this context, a randomized, double-blinded, Placebo-controlled phase II clinical study was conducted to investigate the efficacy of the combination therapy involving platinum-etoposide, the anti-PD-L1 antibody Atezolizumab, and the novel drug Trilaciclib in patients with advanced-stage SCLC (NCT03041311) [62,63]. The trial included 100 treatment-naive advanced SCLC individuals. These patients were randomly assigned to two groups: one receiving Trilaciclib in combination with etoposide/carboplatin/Atezolizumab (E/P/A), and the other receiving Placebo in combination with E/P/A. The groups exhibited similar levels of efficacy in terms of their anti-tumor results. The usage of Trilaciclib compared to Placebo led to the production of a higher number of newly expanded peripheral T cell clones. This expansion was particularly pronounced among patients who exhibited an anti-tumor response to the treatment. In comparison to the administration of Placebo, the administration of Trilaciclib prior to E/P/A resulted in an enhanced patient experience in receiving therapy for ES-SCLC. This was evidenced by a decrease in myelosuppression, as well as the betterment of the health-related quality of life (HRQoL) and safety profiles. Nevertheless, there was no crucial difference observed in the median OS between the two groups. Hence, Trilaciclib demonstrates significant promise as a potential new benchmark for providing supportive treatment to SCLC patients who are undergoing myelosuppressive chemotherapy. 

### 5.2. WEE1

**Figure 3:**
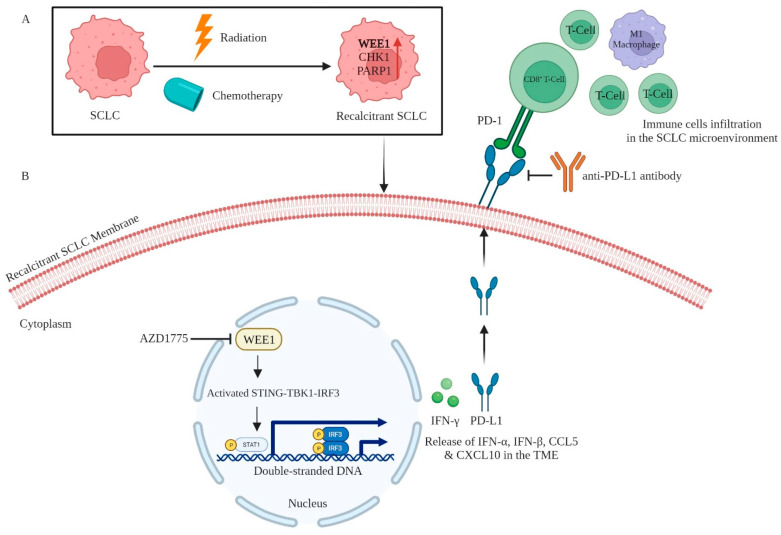
WEE1 kinase signaling, and inhibition involved in SCLC immunotherapy. (**A**) Treatment of SCLC with chemotherapeutic drugs eventually leads to acquired resistance to chemotherapeutics. Cancer cells resist chemotherapy-induced DNA damage by overexpressing DNA repair proteins, including WEE1. (**B**) The pharmacological inhibitor of WEE1 (AZD1775) activates the STING–TBK1–IRF3 pathway to promote PD-L1 expression on the surface, which sensitizes SCLC to anti-PD-L1 antibodies. [Created with BioRender.com (accessed on 18 February 2024)].

WEE1, a kinase responsible for regulating the G2/M checkpoint in response to the damaged DNA, has been observed to be elevated in SCLC. WEE1 primarily modulates cell-cycle progression by phosphorylating CDK1 and subsequently inhibiting its activity. The sensitization of ovarian, colon, cervical, osteosarcoma, glioblastoma, and lung cancer cells to DNA damage caused by irradiation and topoisomerase inhibition has been reported with WEE1 inhibition [64,65,66]. The WEE1 inhibitor AZD1775 (also known as MK-1775) was identified via a high-throughput screening of a chemical compound library of drugs [67]. Analyzing its activity against a panel of 223 kinases identified 8 kinases. Among these 8 kinases, WEE1 inhibition was 10-fold higher compared to the other 7 kinases with 1 μmol/L of AZD1775 in in vitro kinase assays. It inhibits WEE1 kinase in an ATP-competitive manner. In SCLC, mechanistically inhibiting WEE1 using AZD1775 activates the STING (stimulator of interferon genes)-TBK1 (TANK-binding kinase 1)-IRF3 (interferon regulatory factor 3) pathway [19]. This activation leads to an increase in type I interferons (IFN-α and IFN-β) and pro-inflammatory chemokines (CCL5 (C-C chemokine ligand 5) and CXCL10 (C-X-C chemokine ligand 10)), which in turn facilitates immune responses through the infiltration of CD8^+^ cytotoxic T cells. Concomitantly, the inhibition of WEE1 results in the activation of the STAT1 (signal transducer and activator of transcription 1) pathway, leading to an upregulation of IFN-γ and PD-L1 expression, sensitizing its blockade using anti-PD-L1 antibodies. Therefore, simultaneously inhibiting WEE1 and PD-L1 has been reported to induce significant regression of SCLC tumors. However, AZD1775 also poses adverse effects in patients, including anemia and thrombocytopenia. Inhibition of WEE1 using AZD1775 also leads to the activation of type I and II interferon pathways, as well as the infiltration of several immune cell populations, including CD3^+^ and CD8^+^ CTLs (cytotoxic T lymphocytes), CD44^+^ effector/memory T cells, and M1-phenotype macrophages (pro-inflammatory or anti-tumor) in the TME of SCLC. In line with this, the study presented in a Mini Oral Session at the ESMO (European Society for Medical Oncology) Targeted Anticancer Therapies Congress 2022 [68] demonstrated significant tumor regression in mouse SCLC models when AZD1775 and anti-PD-L1 inhibition were combined. Surprisingly, the U.S. FDA has not yet granted approval for the utilization of this medication in the management of any known medical condition. In addition, AZD1775 has undergone assessment as a standalone treatment for advanced SCLC in a phase I clinical trial registered under the identifier NCT02482311 [69]. The efficacy of this treatment has been evaluated in many solid tumors, including breast cancer and metastatic colorectal cancer (mCRC), both as a standalone therapy and in conjunction with other treatments. Overall, chemotherapy induces WEE1 expression in cancer cells to promote PD-L1-mediated T cell suppression (Figure 3).

### 5.3. CHK1

Like WEE1, CHK1, another kinase involved in cell cycle checkpoint regulation, is likewise upregulated in response to damaged DNA and has a pre-eminent role in the cellular responses to DNA damage repair (DDR) generated by chemotherapeutic agents. A human SCLC cell line-based study showed that the SCLC cells acquire resistance to the CHK1 inhibitor Prexasertib (LY2606368) by overexpressing WEE1, leading to a faster DDR [17]. The reversal of this resistance was observed through the use of WEE1 siRNA (short interfering RNA or silencing RNA) or through the inhibition of WEE1 [17].

A Prexasertib monomesylate monohydrate derivative has been synthesized to inhibit CHK1 activity [70,71]. It attenuates CHK1 activity in cell-free assays at IC_50_ < 1 nM and induces DNA damage. However, its limitation is its low oral bioavailability. This derivative was further improvised to generate Prexasertib monolactate monohydrate, which demonstrated enhanced aqueous solubility [72].

**Figure 4:**
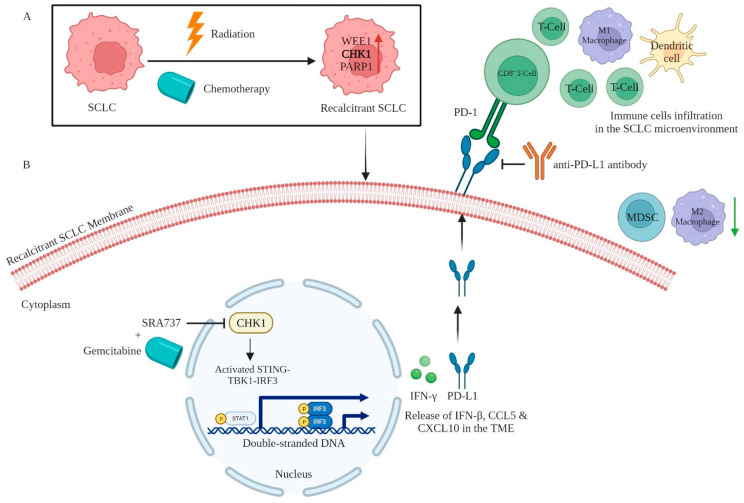
CHK1 kinase signaling, and inhibition involved in SCLC immunotherapy. (**A**,**B**) CHK1, a DNA-repair protein, is overexpressed in SCLC. It participates in the DNA repair process. The combination of the CHK1 inhibitor SRA737 and a low dose of gemcitabine (chemotherapeutic drug) augments the impact of PD-L1 IC blockade (via the use of anti-PD-L1 antibody). This augmentation is achieved by significantly elevating the presence of CD8^+^ T cells, DCs, and M1 macrophages within the TME. [Created with BioRender.com (accessed on 18 February 2024)].

In alternative contexts, it has been documented that CHK1 could elevate PD-L1 levels. This occurs via activation of the STAT1/3-mediated regulation of IRF1 (interferon regulatory factor 1) in NSCLC, prostate cancer, and osteosarcoma [73,74,75]. Nevertheless, the impact of CHK1 on the regulation of PD-L1 expression in SCLC remains uninvestigated. The efficacy of the CHK1 inhibitor Prexasertib has been assessed in a Phase II clinical trial as a standalone treatment for ES-SCLC (NCT02735980) [76,77]. The study described herein represents the initial clinical trial investigating the efficacy and safety of Prexasertib in individuals diagnosed with SCLC [77]. The efficacy of Prexasertib was assessed in two distinct cohorts of SCLC patients: cohort 1, consisting of individuals with platinum-sensitive SCLC, and cohort 2, comprising patients with platinum-resistant SCLC. The study examined a total of 118 participants, with 58 participants in one group and 60 participants in the other group. The primary end point was the objective response rate (ORR). The observed ORR for patients in cohort 1 was determined to be 5.2%, while cohort 2 exhibited a 0% ORR. In cohort 1, 51.7% of patients exhibited progressive disease (PD) as the most favorable treatment response, but in cohort 2, this percentage increased to 61.7%. The secondary end points were the disease control rate (DCR), PFS, and OS. The DCR in cohort 1 was observed to be 31.0%, while in cohort 2, it was found to be 20.0%. The median PFS was shown to be 1.41 months for cohort 1, which consisted of 58 patients with 54 events. Similarly, cohort 2, comprising 60 patients with 60 events, exhibited a median PFS of 1.36 months. The median OS was observed to be 5.42 months for cohort 1, while for cohort 2, it was found to be 3.15 months. In aggregate, the findings indicate that Prexasertib did not exhibit sufficient efficacy as a standalone treatment for ES-SCLC, hence discouraging its further advancement in clinical development. In an alternate situation, the combination of CHK1 inhibition using another inhibitor SRA737 and a low dose of gemcitabine (chemotherapeutic drug) was observed to enhance the impact of PD-L1 IC blockade (via the use of an anti-PD-L1 antibody) in an immunocompetent *Rb/TP53/p130* (*RPP*) tumor-bearing SCLC mouse model [20]. This enhancement was achieved by significantly increasing the presence of CD8^+^ T cells, DCs, and M1 macrophages within the TME. The administration of this treatment protocol resulted in a notable reduction in the populations of immunosuppressive M2-phenotype macrophages (anti-inflammatory or pro-tumor) and MDSCs (myeloid-derived suppressor cells), together with an elevation in the expression levels of the Type I IFN gene, *IFN-β*, and the chemokines CCL5 and CXCL10 inside the TME of SCLC (Figure 4). SRA737 has undergone clinical evaluations both as a standalone treatment and in conjunction with low-dose gemcitabine for advanced solid tumors, including SCLC (NCT02797977).

It is widely recognized that both CHK1 kinase and PARP (non-kinase) are proteins participating in the DDR and are triggered in response to damaged DNA. Upon the occurrence of DNA single-strand breaks (DNA-SSBs), CHK1 is elicited and facilitates the coordination of the DDR through the ATR (ataxia telangiectasia rad3-related)–CHK1 pathway, as well as the activation of cell cycle checkpoint responses in cancer cells [78]. Conversely, PARP assumes a pivotal role in the nucleotide excision repair (NER) and base excision repair (BER) pathways in cancer cells. It permits the repair of DNA damage induced by alkylating agents and chemotherapeutic drugs [79]. Hence, when CHK1 or PARP are inhibited, the vulnerability to DNA damage and cellular death is increased. A study was carried out to examine the effects of CHK1 and PARP inhibitors in vitro using human and murine SCLC cell lines and in vivo using murine RPP/mTmG cells derived from a genetically engineered (GE) SCLC mouse model with the conditional loss of *TP53*, *p130*, and *Rb* (*RPP*). The results of this study were subsequently analyzed [80]. To achieve the intended objective, the use of the CHK1 inhibitor Prexasertib and PARP inhibitor Olaparib (manufactured by KuDOS Pharmaceuticals, Cambridge, UK) was employed. In the context of in vitro experiments, it was observed that targeting the DDR using either the CHK1 inhibitor or the PARP inhibitor in various human SCLC cell lines resulted in an increase in the expression of the PD-L1 protein. This increase was quantified using a Reverse Phase Protein Array (RPPA) [81,82], with the CHK1 inhibitor showing the highest fold-change and the PARP inhibitor demonstrating a noticeable fold-change in PD-L1 expression across the tested cell lines. The findings were validated using immunoblot analysis, which additionally demonstrated an increase in PD-L1 expression following the DDR targeting in a time-dependent fashion. To effectively target PD-L1, it is imperative that it is expressed on the cell surface. Consequently, researchers proceeded to evaluate the expression of PD-L1 on the surface of cells using fluorescence-activated cell sorting (FACS). The PD-L1 expression on the surface of cell exhibited a notable rise in a time-dependent fashion in both human and murine SCLC cell lines upon treatment with either Prexasertib or Olaparib. To ascertain the cause of PD-L1 overexpression, researchers conducted knockdown (KD) experiments targeting *CHEK1* (or *CHK1*) or *PARP* in multiple SCLC cell lines. This was performed to determine whether the observed upregulation was a direct result of CHK1 or PARP inhibition, rather than an unintended consequence of the “off-target” effects caused by the inhibitors. In accordance with pharmacological inhibition, the expression of PD-L1 was significantly elevated in cells with *CHEK1* KD or *PARP* KD, as compared to in the control. The elevation of PD-L1 following *CHK1* targeting was additionally validated by subjecting cells to a second CHK1 inhibitor, LY2603618 (also known as Rabusertib, manufactured by Eli Lilly and Company, Indianapolis, IN, United States), in SCLC cell lines [77].

In the context of in vivo experiments utilizing SCLC *RPP* mouse models, both immunocompromised (i.e., nude) and immunocompetent (i.e., B6129F1) models were employed. The results indicated that the delay in tumor growth induced by Prexasertib was notably more pronounced in the immunocompetent model compared to in the immunocompromised model. These findings yield evidence for the effectiveness of CHK1 targeting when the immune system remains intact. The administration of Prexasertib resulted in the upregulation of PD-L1 protein expression in both the immune-deficient (ID) and immunocompetent in vivo models. In contrast, the immunocompetent model exhibited a higher level of PD-L1 overexpression compared to the ID model. The immunoblot analysis provided further confirmation of the upregulation of the expression of PD-L1 in the immunocompetent model. The researchers also observed that SCLC immunocompetent lung tumors treated with the CHK1 inhibitor exhibited a notable increase in the infiltration of CD3^+^ total T cells and CD8^+^ T cells. However, there was an alleviation in CD4^+^ helper T cells, PD-1^+^/TIM3^+^ (T cell immunoglobulin and mucin domain-containing protein 3 positive) exhausted T cells, and CD62L^+^ naive T cells. Additionally, the treated tumors showed the augmented infiltration of CD44^+^ memory/effector T cells compared to tumors treated with a vehicle. The upregulation of PD-L1 expression, swift regression of tumors, and infiltration of immune cells into tumors after inhibiting CHK1 indicate a direct role for DDR modulation in regulating the immune milieu in these SCLC models. In addition, given that the inhibition of CHK1 alone did not lead to complete tumor eradication, despite observed reductions in tumor development, the enhanced infiltration of T cells, and alleviation of T cell exhaustion in vivo, the researchers proceeded to assess the potential of CHK1 inhibition for sensitizing tumors to PD-L1 blocking in immunocompetent *RPP* mice. When the anti-PD-L1 antibody was administered as a monotherapy, the mice did not exhibit any discernible anti-tumor response and were subsequently euthanized due to the development of an overwhelming tumor load within a period of three weeks. Nevertheless, a noteworthy reduction in tumor size was found when the CHK1 inhibitor Prexasertib and anti-PD-L1 antibody were administered in combination. Hence, the suppression of CHK1 resulted in an enhanced anti-tumor immune response elicited by the anti-PD-L1 antibody. The augmentation of anti-tumor immunity elicited by the anti-PD-L1 antibody was further enhanced through the suppression of PARP. The study indicated that the anti-tumor immune responses following DDR targeting were observed to be mediated through the STING–TBK1–IRF3 pathway in SCLC, which stimulated secretion of the chemokines CCL5 and CXCL10 [80]. This eventually led to the activation and function of CTLs. Collectively, these findings illustrate the notable effectiveness of the concurrent use of PD-L1 blocking with CHK1 or PARP inhibition, thereby establishing a robust scientific justification for the integration of these approaches in clinical studies involving individuals with SCLC. In this regard, Thomas et al. undertook a clinical trial (NCT02484404) to evaluate the effectiveness of the combination of Olaparib and Durvalumab (antiPD-L1 antibody) in a cohort of 20 patients with refractory ES-SCLC [83]. Out of the 19 patients who were eligible for evaluation, a mere 2 individuals, accounting for 10.5% of the sample, exhibited complete response (CR) or partial response (PR) to the administered treatment. Additionally, the median PFS duration was found to be 1.8 months. In a separate phase II study (NCT02734004) involving 38 patients with relapsed SCLC [84], Krebs et al. presented preliminary findings in abstract format. The study indicated that the combination therapy of Olaparib and Durvalumab was typically well-endured, with two patients achieving a confirmed PR or CR. However, the primary objective of DCR, which includes CR, PR, and stable disease, at the 12-week mark, was found to be 29%, falling within the futility region. A supplementary phase I clinical trial (NCT02660034) was conducted to evaluate the safety of combining the PARP inhibitor Pamiparib (also known as BGB290, manufactured by BeiGene, Beijing, China and Cambridge, MA, United States), Basel, Switzerland) with the anti-PD-1 antibody Tislelizumab (also known as BGB-A317) in 49 patients with recurrent SCLC [85]. The participants enrolled in the trial received a minimum of one administration of either Pamiparib or Tislelizumab. A total of four patients exhibited dose-limiting toxicities. Nausea was reported as the most observed treatment-emergent adverse event, occurring in 63% (31 out of 49) of patients. Hepatitis, namely autoimmune hepatitis, was the sole significant adverse event observed in two or more individuals, accounting for 8% (4 out of 49 patients). After a median follow-up period of 8.3 months, a total of 10 patients out of 49 (20%) had an ORR based on the Response Evaluation Criteria in Solid Tumors (RECIST) version 1.1. This included two patients with CR and eight patients with PR.

### 5.4. Macrophage-Mediated Anti-Cancer Therapies

It has been reported that tumor-associated (or tumor-infiltrated) macrophages (TAMs) present in the tumor microenvironment (TME) express IC protein PD-1 (i.e., PD-1^+^ TAMs) in both mouse and human colon cancer cells [58]. The expression of PD-1 is augmented with advancing time and disease stages in mice and human colon cancer cells, respectively. The expression of PD-1 on TAMs hinders their phagocytic activity against tumor cells. In vivo, the blockade of PD-1/PD-L1 enhances the process of macrophage phagocytosis, attenuates the growth of tumors, and extends the lifespan of mice with cancer in a manner that is macrophage-dependent. However, ambiguity exists as one of the studies on SCLC involving immunohistochemistry (IHC) and RNA-sequencing analysis revealed that TAMs also express the IC protein PD-L1 (i.e., PD-L1^+^ TAMs) [86], which attenuates the phagocytosis of SCLC cells through its interaction with its cognate receptor PD-1 expressed on immune cells, such as T cells. This facilitates SCLC growth and spread. Therefore, monoclonal antibodies (mAb; or ICIs) targeting either PD-L1 or PD-1 biomarkers are one of the therapeutic approaches to combat SCLC.

An open-label, multi-drug, phase II clinical trial (NCT02937818) was carried out in patients with refractory ES-SCLC who did not progress from platinum-based chemotherapy. The trial enrolled 72 patients and six therapeutic interventions, namely Durvalumab (anti-PD-L1 antibody), Tremelimumab (anti-CTLA-4 antibody), AZD1775 (WEE1 inhibitor), Olaparib (PARP inhibitor), AZD6738 (ATR inhibitor), and carboplatin (chemotherapy). These therapeutic interventions were evaluated in different combinations. Patients were randomly divided into the following treatment arms: Durvalumab plus Tremelimumab (arm A), AZD1775 plus carboplatin (arm B), Olaparib plus AZD6738 (arm C). The primary end point was ORR, whereas PFS and OS were the secondary end points. The ORR in arm A was 9.5%, in arm B, it was 0.0%, whereas in arm C, it was 4.8%. The PFS in arms A, B, and C was 1.91, 2.60, and 2.92 months respectively. The OS for the three treatment arms was 5.95 months, 4.67 months, and 7.56 months, respectively. Another PARP inhibitor, Fluzoparib (also known as SHR-3162, manufactured by Jiangsu Hengrui Medicine Co Ltd., Lianyungang, China), is currently being evaluated in concert with an anti-PD-1 antibody in an open-label, phase II clinical trial (NCT04782089) in patients with ES-SCLC who did not improve after first-line chemotherapy. Table 3 summarizes the protein kinase (PK) inhibitors being tested in different clinical trials for SCLC.

## 6. Repurposing Protein Kinase and Other Inhibitors against SCLC

The limited therapeutic benefits observed with available kinase inhibitors alone or in combination with approved therapies has urged clinicians to develop additional therapies. Repurposing existing therapeutic drugs is an attractive drug development strategy, which is quicker, cheaper, and safer. Modern computational drug screening methods and proteo-genomic discovery methods have allowed for the identification of drugs that are approved for other diseases but hold the potential to target malignancies as well. On this note, below we summarize the kinases that are dysregulated in SCLC and their clinically approved inhibitors, which can be used against SCLC.

One of the common kinases that is hyperactive in transformed malignant cells is PI3K [87]. Recent advances in phospho-proteomic technologies have allowed us to identify hyperactive kinases in SCLC. It is noteworthy to mention here that PI3K signaling is significant in many immune cell types such as CD4, CD8, and T_regs_ therefore the immunomodulatory traits of inhibitors of PI3K are also pivotal in the context of hematological malignancies and other cancers. Having said that, in one of the reports on chronic lymphocytic leukemia (CLL) [88], the investigators evaluated the impact of three clinically available PI3Kδ inhibitors, namely Idelalisib (CAL-101), Duvelisib (IPI-145), and Umbralisib (TGR-1202), on T_regs_. This investigation encompassed in vitro experiments conducted on normal human T cells, T cells derived from patients suffering from CLL, and T cells within an *Eμ-TCL1* adoptive transfer (transgenic) mouse model simulating CLL in vivo. This study, carried out by Maharaj et al. [88], aimed to examine the potential impact of CK1ε (casein kinase 1 epsilon) blockade using Umbralisib in conjunction with Idelalisib and Duvelisib on T_reg_ effects in healthy human T cells and T cells derived from CLL patients. According to reports, the inhibition of CK1ε resulted in an increase in T_regs_ in patients with CLL [88]. As for Umbralisib, it downregulates the Wnt pathway through the inhibition of CK1ε in CLL, while also demonstrating reduced negative impacts on the immunosuppressive capabilities of T_regs_. Although there have been no selective CK1ε inhibitors that have progressed to the clinical stage thus far, a compound known as Umbralisib, which inhibits both CK1ε and PI3Kδ, has been investigated in Phase II clinical studies involving patients with CLL and non-Hodgkin lymphoma (NHL) [89]. In the Phase II clinical trial (NCT04163718), treatment-naive patients were administered Umbralisib (TGR-1202) as a monotherapy for CLL [90]. However, due to the observed severe toxicity, Umbralisib was withdrawn by the FDA. Moreover, publicly available data indicate that CK1ε is specifically overexpressed in SCLC patient tumors compared to other types of lung cancers and normal lungs [91]. Increased PI3K and CK1ε activity may provide a survival and proliferation advantage to the SCLC cells and may act as suitable drug target. However, careful drug-dose optimization and proper selection of the patient subgroup is required to explore the possibility of repurposing PI3K and CK1ε inhibitors against SCLC.

The SCLC tumor is classified as a high-grade NE neoplasm. According to reports, the activity of transcription factors has been associated with the transition from NE-high-variant SCLC to NE-low-variant SCLC. The transcription factor known as MYC is an example of such a factor. According to reports, the amplification of the members of the *MYC* family, namely *MYC*, *MYCN*, and *MYCL* (which are paralogs), has been linked to phenotypic variations in SCLC. This amplification has been found to promote a specific subtype of SCLC known as the NE-low-variant subtype [92]. The carcinogenesis process is initiated by evading many processes that act as checkpoints to restrict tumor growth. These mechanisms include the arrest of cell multiplication, induction of cell death, and/or triggering of cellular senescence. In addition, the susceptibility of SCLC cells to AURK inhibitors (AURKi) is determined by members of the MYC family. According to the literature, SCLC cells with *MYC* amplification have been found to have sensitivity to AURKi, Alisertib (AURK-A inhibitor), and Barasertib (AURK-B inhibitor) [42,93]. Additional research has indicated that *MYC*-amplified SCLC may exhibit increased sensitivity to the inhibition of CHK1 [76]. The activation of gene transcription by *MYC* in combination with MAX (MYC-associated factor X), a transcription factor, is well documented and occurs through multiple methods. This phenomenon facilitates the growth and proliferation of SCLC cells. Hence, the utilization of MYC family members as biomarkers for the purpose of predicting therapeutic vulnerability in SCLC can be justified. However, one study was conducted n SCLC [94], where MYC inhibition using Omomyc (MYC/MAX inhibitor) was reported to suppress the growth of tumors with *Rb* and *TP53* inactivation. Mechanistically, Omomyc induced arrest of the cell cycle in the G1 phase and/or apoptosis in SCLC cells. G1 cell cycle arrest mediated by Omomyc was dependent on the excitation of CDKN1A (CDK inhibitor 1A, also known as p21), in part through TP73 (and important E2F1 apoptotic target gene in the DDR pathway). Studies reported that prolonged tumor regression was achieved in wild-type animals through the suppression of *MYC* and several other oncogenes in GE conditional mouse models of lymphoma/leukemia [95,96,97,98]. Nevertheless, when *MYC* was suppressed in the host with a compromised immune system, there was a reduced rate of elimination of tumor cells, the partial shrinkage of tumors, and the eventual recurrence of tumors [99,100,101]. The persistent reduction of tumors caused by *MYC* inactivation was prevented only when CD4^+^ T cells were absent [102]. In conclusion, it was demonstrated that the inhibition of *MYC* primes the tumor for CD4^+^-mediated anticancer activities. A similar mechanism may also exist in SCLC.

In addition to the DDR response to chemotherapeutics, there is an increasing scholarly focus on elucidating kinase-specific somatic mutations that are correlated with SCLC. The *RET* (rearranged during transfection) proto-oncogene, which pertains to the receptor tyrosine kinase (RTK) family, has been found to incorporate somatic mutations in cases of SCLC [102]. Metastatic SCLC tumors have been found to exhibit activating M918T *RET* somatic mutations, which have been confirmed using Sanger sequencing. Previous studies have documented that the sustained overexpression of both mutant M918T and wild-type (WT) *RET* in SCLC cell lines results in the excitation of MAPK/ERK (mitogen-activated protein kinase/extracellular signal-regulated kinase) signaling, upregulation of MYC expression, and enhanced cellular proliferation, with mutant RET exhibiting a more pronounced effect. The stable cells exhibited an increased sensitivity to the RET tyrosine kinase inhibitors (TKIs) Vandetanib and Ponatinib. An additional investigation into the expression of RET mRNA (messenger RNA) in SCLC has unveiled considerable heterogeneity in both individual cells and tumors. Notably, SCLC cells have exhibited markedly elevated levels of *RET* expression in comparison to lung adenocarcinoma cells, which belong to the NSCLC category [103].

In the context of the immune system, it has been reported that the signaling pathway involving RET in monocytes leads to an increase in the expression of immunosuppressive cytokines and chemokines [104]. Tumors have been observed to selectively attract MDSCs to hinder immunological responses and impede the effectiveness of immunotherapy. Hence, if the signaling by RET within the tumor cells is impeded, the tumors will fail to facilitate the infiltration of MDSCs, resulting in immune activation and subsequently eliciting an immunotherapeutic response. An analysis of SCLC primary tumor samples using a panel of oncogenic mutations has identified an activating M918T RET somatic mutation [103]. Nevertheless, the efficacy of RET inhibitors has been assessed in SCLC. The efficacy of the RET inhibitor Vandetanib was assessed in an SCLC phase II clinical trial carried out by the National Cancer Institute (NCI) of Canada Clinical Trials Group Study BR.20 [105]. According to the experiment, a total of 100 eligible patients who had achieved either a CR or PR after combination chemotherapy (specifically thoracic or prophylactic cranial irradiation, PCI) were administered either oral Vandetanib or a Placebo. The median PFS for patients receiving Vandetanib was 2.7 months, while for those receiving the Placebo, it was 2.8 months. The observed OS for Vandetanib was found to be 10.6 months, while the OS for the Placebo group was 11.9 months. The trial concluded that Vandetanib did not demonstrate effectiveness as a “maintenance therapy” for SCLC.

Among the non-RTKs (NRTKs), FAK (focal adhesion kinase) has been reported to be amplified and upregulated in SCLC tumors [106,107,108,109] and activated in SCLC cell lines [106]. Based on the aforementioned data, it was postulated by researchers that the activation of FAK in SCLC has a role in its aggressive nature, hence suggesting FAK as a potential therapeutic target for SCLC [110]. This study showed that the FAK pharmacological inhibitors (specifically PF-573228, PF-562271, and FAK Inhibitor 14) suppressed the activity of FAK by reducing the levels of phospho-FAK (Tyr397 or Y397), while not affecting the overall expression of total FAK. Moreover, the compound PF-573228 exhibited a reduction in cellular proliferation and DNA synthesis, as well as the induction of cell cycle arrest specifically in the G2/M phases. Additionally, it was shown that PF-573228 promoted apoptosis across all tested cell lines. Besides as a single-agent, FAK inhibitors have also been used in combination therapies, to improve the chemotherapy, targeted therapy, and immunotherapy clinical outcomes [111].

It has also been documented that FAK has an important role in the evasion of cancer cells from immune surveillance through various methods. One study has elucidated the role of FAK in regulating the levels of T_regs_ in cutaneous and pancreatic tumors [109,112]. A study conducted on cutaneous squamous cell carcinoma (SCC) revealed that FAK plays a significant role in the infiltration of and increase in T_reg_ levels within the tumor. This eventually impeded the anti-tumor response mediated by CD8^+^ CTLs [112]. Hence, it was shown that the pharmacological inhibition of FAK in murine models of cutaneous SCC led to the attenuation in T_reg_ levels and augmentation in CD8^+^ CTL levels. This finding provides a confirmation of the crucial involvement of FAK in the immune evasion mechanism employed by cancer cells. Comparable findings were also noted in pancreatic ductal adenocarcinoma (PDAC) and colorectal cancer (CRC), wherein the combination of FAK inhibitors and immunotherapy resulted in significantly enhanced survival outcomes in murine models [10,113]. In addition, previous studies have documented the ability of FAK inhibitors to decrease the presence of immunosuppressive cells that infiltrate tumors in both pancreatic [10,114] and breast cancers [115]. In the context of SCC, FAK TKIs have demonstrated efficacy in enhancing tumor management by reducing the presence of tumor-infiltrating T_reg_ cells and surging the infiltration of CD8^+^ CTLs [112]. Moreover, past studies have demonstrated that FAK facilitates the upregulation of IL-33, which binds to sST2 (soluble suppressor of tumorigenicity 2) protein, as well as CCL5 in cells of skin SCC. Hence, it has been demonstrated that IL-33 and ST2 have a role in facilitating FAK kinase-dependent mechanisms involved in SCC immune evasion [109,116]. It is possible that such pathways may also exist in SCLC.

Inhibitors of FAK have been evaluated in clinical trials for various malignancies. The FAK inhibitor VS-6063 (also known as Defactinib) was evaluated in a phase I clinical trial of pancreatic neoplasms (NCT02546531) [117]. The combination agent was Pembrolizumab (anti-PD-1 antibody). Out of the 20 patients who were treated for refractory PDAC, the median PFS was 3.6 months, and the median OS was 7.8 months. The concurrent administration of Defactinib, Pembrolizumab, and Gemcitabine demonstrated favorable tolerability and safety, exhibited encouraging initial effectiveness, and displayed biomarker activity in infiltrative T cells. Therefore, FAK inhibitors hold considerable potential to be repurposed against SCLC.

Gene co-expression network methodologies have emerged as a pivotal field of research. These approaches have demonstrated efficacy in elucidating gene modules that play a pre-eminent role in driving phenotypic features across various biological systems, encompassing diverse cancer types. One study was performed to investigate the role of spleen tyrosine kinase (*SYK*) as an oncogenic driver in SCLC using weighted gene co-expression network analysis (WGCNA) based on lung cancer datasets [118]. This presents the possibility of considering SYK as an additional therapeutic alternative for the treatment of SCLC. The activation of SYK is known to initiate a series of subsequent cellular processes that facilitate the survival and spread of cells. These processes include the activation of PI3K and AKT (also known as PKB (protein kinase B)), as well as the phosphorylation of other signaling proteins. These findings have been documented in CLL [119] and acute myelogenous leukemia (AML) [120]. The present study found that the use of *SYK* siRNA resulted in the attenuation of the rate of proliferation and an augmentation in cell death in SCLC cell lines that expressed *SYK* [118].

In addition to cell proliferation, the hyperactivation of SYK activates aberrant B cell signaling in CLL. This signaling occurs through a physical interaction between SYK and the immunoreceptor tyrosine-based activation motif (ITAM) present in the B cell receptor (BCR) complex. This interaction has been found to have a positive effect on the survival and proliferation of B cells during their development and immune response [121]. In contrast to this, SYK activation through the Fc receptors of B cells has been observed to induce apoptosis in DLBCL (diffuse large B cell lymphoma) [122,123]. B cells undergo apoptosis in response to *SYK* KD and exhibit impaired development in vivo in mouse models lacking SYK expression [123]. The aforementioned data collectively provide a justification for the utility of small-molecule inhibitors of kinases as a therapeutic approach targeting SYK in hematological malignancies, namely in AML [120], DLBCL [124], and in NHL and CLL [125]. The investigation of SYK in relation to lung NE cells and the cells of genesis for SCLC, however, remains unexplored in the existing literature.

## 7. Protein Kinases in Personalized Medicine for SCLC

SCLC was considered a single disease for many years. However, the availability of modern technology to sequence transcriptomes allowed for the classification of SCLC molecularly [126].

Initial studies with SCLC patient tissues and cell lines identified a “classic” or “variant” type of SCLC, where the “classic” type was associated with NE features, while these features were low or absent in the “variant” type [127].

Using transcriptomic analysis, various studies from different labs identified distinct molecular subtypes of SCLC [126,128,129,130]. Owonikoko et al. showed four discrete subtypes of SCLC defined by variation in the expression levels of the transcription factors *ASCL1* (SCLC-A), *NEUROD1* (SCLC-N), *POU2F3* (SCLC-P), and *YAP1* (SCLC-Y) [129]. Among these subtypes, the *YAP1*-positive subtype was associated with a higher immune signature and prolonged patient survival compared to other subtypes [129]. Another transcriptomic study performed by Gay et al. confirmed SCLC-A, -N, and -P subtypes [128]. However, they did not identify the SCLC-Y subtype, rather the SCLC-Inflamed subtype (SCLC-I). The existence of the SCLC-Y subtype was also not confirmed by another single-cell transcriptomic study conducted by Chan et al. [48]. The NE gene signature analysis identified that the SCLC-A and SCLC-N are NE-high, whereas SCLC-P and SCLC-Y (or SCLC-I) are NE-low. Overall, these studies have identified four distinct subgroups of SCLC patients who are vulnerable to different types of therapeutic regimes. Reportedly, the addition of immunotherapy to chemotherapy provided the most significant advantage for SCLC-I. On the other hand, the remaining subtypes demonstrated specific therapeutic vulnerabilities; for example, SCLC-P was susceptible to PARP inhibitors, SCLC-N to AURK inhibitors, or SCLC-A to BCL-2 (B-cell leukemia/lymphoma, an apoptosis regulator protein) inhibitors. Another study by Mollaoglu et al. identified the switching of SCLC-A to SCLC-N that was driven by the overexpression of *MYC* [42]. This subtype switching generated distinct sensitivity to AURK inhibitors, specifically Alisertib (an inhibitor of Aurora A) and Barasertib (an inhibitor of Aurora B) [42]. This sensitivity was observed to significantly augment the response to chemotherapy in vivo. Hence, the study suggests that the utilization of AURK inhibition alongside first-line chemotherapy (cisplatin-etoposide) may act as a feasible therapeutic approach against *MYC*-driven SCLC. Therefore, researchers proposed aligning the initial tumor subtype with the appropriate treatment to personalize therapy and improve the extent and duration of the response in SCLC patients.

## 8. Conclusions

Our understanding of the molecular mechanisms of SCLC has been limited for decades due to the unavailability of animal models mimicking human SCLC characteristics. The development of an SCLC transgenic mouse model has provided a significant tool to identify the driver genes and discover new therapeutic vulnerabilities in SCLC. The insights generated using this model led to the clinical trials evaluating a CHK1 inhibitor (i.e., LY2606368) and PARP inhibitor (i.e., Olaparib). These studies showed that combining kinase inhibitors with ICIs further improves immune responses and survival, following the infiltration of immune cells (mainly CD8^+^ T cells) in the SCLC microenvironment. These observations clearly show the importance of targeting multiple pathways simultaneously to tackle the heterogeneous SCLC disease. Although transgenic mouse models mimic human SCLC, further preclinical studies with PDX and humanized mouse models are required to predict the therapeutic potential of innovative therapies.

The development of targeted therapies using kinase inhibitors has revolutionized the therapeutic regime for cancer treatment. A significant clinical benefit has been observed with the use of small-molecule kinase inhibitors over conventional chemotherapies. However, these benefits are marginal in SCLC. Modern single-cell genomic and multiplex spatial proteomic studies have established the highly heterogeneous nature of SCLC tumors, which is a major factor responsible for the limited benefits of targeted therapies observed in SCLC.

Therefore, a therapeutic strategy combining multiple drug targets may be required to treat heterogenous SCLC. This approach was proven to be beneficial wherein combining a dual chemotherapy regime with additional DDR inhibitors or immunotherapy improved patients’ survival. These observations will urge clinicians to carefully utilize unique therapeutic vulnerabilities alone or in combination with therapies in the subgroups of SCLC patients to obtain improved clinical responses.

Emerging phospho-proteomic technologies and modern computational methods have allowed us to align the dysregulated molecular signatures in SCLC with the other pathological molecular signatures and identify the clinically approved drugs associated with these molecular signatures. This analysis has unraveled the possibility of repurposing the drugs approved for other diseases against recalcitrant and metastatic SCLC. Drug repurposing has emerged as a pivotal tool in drug discovery as it allows for an exploration of the additional applications of existing drugs, a higher probability of success, a faster path to reach the clinic and lower costs associated cancers with repurposed drug development, and most importantly, increases in the portfolio of available effective cancer chemotherapeutic agents for patients [110]. Additionally, it is important to acknowledge the current limits, such as the transition towards the utilization of large-scale repurposing methodologies, leveraging the growing capabilities of data analysis through computational repurposing, and the use of repurposed medication candidates, which can be enhanced through the implementation of combination therapy. In conclusion, pharmacological inhibitors against dysregulated kinases have emerged as promising drug targets against SCLC; however, additional preclinical studies are required to validate their therapeutic potential alone or in combination with ICIs.

## Figures and Tables

**Table 1:** Driver genes that are involved in SCLC progression.

Serial No.	Gene(s)	Contribution in SCLC Progression	Reference(s)
1	*Rb* and *TP53*	Inactivation of *Rb* and *TP53* genes in a conditional mouse model induces SCLC.	[24]
2	*PTEN*	Inactivation of one allele of *PTEN* in *Rb/TP53*-deleted mouse model leads to the progression of SCLC.	[47]
3	*CREBBP*	The deletion of *CREBBP* accelerates growth in the *Rb/TP53/Crebbp* autochthonous mouse model.	[34]
4	*Rbl2*	The deletion of this gene results in augmented cell division and a notable increase in the progression of SCLC in vivo, in the *Rb/TP53/Rbl2* (*RPR2*) mouse model.	[37]
5	*MYC*	*MYC* amplification leads to the promotion of aggressive, highly metastatic, and refractory SCLC tumors that are initially responsive to chemotherapy. This effect has been observed in vivo in the *Rb/TP53/Myc*-mutant (*RPM*) mouse model.	[42]
6	*EP300*	A mutated *EP300* gene within the *RPR2* and *RPM* models accelerates SCLC growth, while the complete KO of *EP300* suppresses SCLC growth. A mechanistic study showed the tumor-suppressive role of the HAT domain of the EP300 protein, whereas other domains (i.e., KIX, BAD, and TAZ) showed tumor-promoting activity.	[36]
7	*FGFR1*	In vivo, within the *RP-Fgfr1* mouse model, *FGFR1* demonstrates a context-dependent impact. The impairment of SCLC formation from CGRP^POS^ NE cells is observed. Conversely, it is noted that it promotes the growth of SCLC and low-grade NE bronchial lesions from tracheobronchial-basal cells.	[46]
8	*PLCG2*	Using clinical samples and in vivo models, *PLCG2* has been reported to be associated with higher SCLC metastatic potential, thereby emerging as a potential driver of SCLC progression.	[48]

**Table 2:** List of clinical trials in phase III for treatment-naïve ES-SCLC patients.

S. No.	Clinical Trial Identifier (Name)	Treatment Group	Primary End Point(s)	Outcome	Reference(s)
1	NCT02763579 (IMpower133)	Group I (Treatment): Atezolizumab (PD-L1 inhibitor) + carboplatin-etoposide (C/E) chemotherapy Group II (Control): Placebo + C/E chemotherapy	PFS and OS	PFS: Group I: 5.2 months Group II: 4.3 months OS: Group I: 12.3 months Group II: 10.3 months	[10,11,12,13]
2	NCT03043872 (CASPIAN)	Group I (Treatment): Durvalumab (PD-L1 inhibitor) + carboplatin-etoposide (C/E) chemotherapy Group II (Control): C/E chemotherapy	PFS and OS	PFS: Group I: 5.1 months Group II: 5.4 months OS: Group I: 13.0 months Group II: 10.3 months	[50,51]
3	NCT03066778 (KEYNOTE-604)	Group I (Treatment): Pembrolizumab (PD-1 inhibitor) + carboplatin-etoposide (C/E) chemotherapy Group II (Control): Placebo + C/E chemotherapy	PFS and OS	PFS: Group I: 4.5 months Group II: 4.3 months OS: Group I: 10.8 months Group II: 9.7 months	[52]
4	NCT03711305 (CAPSTONE-1)	Group I (Treatment): Adebrelimab (PD-L1 inhibitor) + carboplatin-etoposide (C/E) chemotherapy Group II (Control): Placebo + C/E chemotherapy	OS	OS: Group I: 15.3 months Group II: 12.8 months	[53]
5	NCT04063163 (ASTRUM-005)	Group I (Treatment): Serplulimab (PD-1 inhibitor) + carboplatin-etoposide (C/E) chemotherapy Group II (Control): Placebo + C/E chemotherapy	OS	OS: Group I: 15.4 months Group II: 10.9 months	[54]

**Table 3:** A list of ICI clinical trials with their protein kinase (PK) inhibitors for SCLC.

PK Inhibitor	Main Target	Role in Immunotherapy in SCLC	Clinical Trial Identifier	Reference(s)
Trilaciclib (G1T28)	CDK4/6	Has a pivotal role in governing the advancement of the cell cycle in cancer cells. The administration of chemotherapy to patients with SCLC leads to an increased peripheral lymphocyte count and improved activation of T cells when CDK4/6 is inhibited. Furthermore, it has been observed that it augments the levels of PD-L1 expression, hence sensitizing its blockage, in syngeneic mouse models conducted in vivo.	NCT03041311	[57], [62,63]
AZD1775 (Adavosertib)	WEE1	Regulates cell cycle checkpoint at G2/M transition in cancer. The inhibition of WEE1 is linked to the surged infiltration of CD8^+^ T cells, facilitating immune responses. Concurrently, this inhibition also elevates IFN-γ and PD-L1 (sensitizing its blockade), via STAT1 activation in in vivo SCLC mouse models.	NCT02937818	[19]
SRA737 Prexasertib (LY2606368) Rabusertib (LY2603618)	CHK1	Involved in the regulation of the cell cycle in cancer. The inhibition of CHK1 augments the effect of PD-L1 blockade in an *RPP* tumor-bearing immunocompetent SCLC mouse model by significantly augmenting the infiltration of CD8^+^ T cells, DCs, and M1-like macrophages (pro-inflammatory/anti-tumor), in the TME		[20] [80]
Olaparib Pamiparib (BGB290) Fluzoparib (SHR-3162)	PARP (non-kinase)	A non-kinase involved in the DDR in cancer. The inhibition of PARP is also associated with the increased infiltration of CD8^+^ T cells in the TME and elevated expression of PD-L1 (sensitizing its blockade) in an *RPP* tumor-bearing immunocompetent SCLC mouse model.	NCT02484404 NCT02734004 NCT02660034 NCT02937818 NCT04782089	[82] [83] [84] [80] [85]

## Data Availability

The data presented in this study are available in this article.
